# Phenolic, Polysaccharide, and Color Variability in Chilean Commercial Chardonnay Wines: Influence of Closure Type, Geographic Origin, and Vintage

**DOI:** 10.3390/foods15101735

**Published:** 2026-05-14

**Authors:** Alvaro Peña-Neira, Karinna Estay, Carla Jara, Manuel Flores-Cabrales, Cristina Ubeda, Mariona Gil-i-Cortiella

**Affiliations:** 1 Department of Agro-Industry and Enology, Facultad de Ciencias Agronómicas, Universidad de Chile, Santa Rosa 11315, La Pintana, Santiago 8820000, Chile; karinna.estay@uchile.cl (K.E.); carlajara@u.uchile.cl (C.J.); 2 Department of Management and Rural Innovation, Facultad de Ciencias Agronómicas, Universidad de Chile, Santa Rosa 11315, La Pintana, Santiago 8820000, Chile; manuelflores@uchile.cl; 3 Área de Nutrición y Bromatología, Departamento de Nutrición y Bromatología, Toxicología y Medicina Legal, Facultad de Farmacia, Universidad de Sevilla, 41012 Sevilla, Spain; c_ubeda@us.es; 4 Departament de Bioquímica i Biotecnologia, Facultat d’Enologia de Tarragona, Universitat Rovira i Virgili, C/Marcel.li Domingo 1, 43007 Tarragona, Spain

**Keywords:** phenolic compounds, polysaccharides, CIELab color, hydroxycinnamates, flavonols, multivariate analysis, wine composition, wine matrix, geographic origin, winemaking practices

## Abstract

Chardonnay (*Vitis vinifera* L.) is one of the most widely cultivated white grape varieties in Chile, yet integrated studies addressing phenolic composition, polysaccharides, and color in commercial wines remain limited. This study characterized 30 commercial Chardonnay wines from major Chilean regions through a comprehensive analysis of phenolic composition, polysaccharide fractions, and CIELab color parameters, considering multiple sources of variability including vintage (2023–2024), closure type, geographic location, and valley of origin. Basic oenological parameters showed low variability, confirming their strong technological regulation in commercial wines. In contrast, total tannins, selected chromatic coordinates (particularly a* and hue angle), polysaccharide fractions, and several low-molecular-mass phenolics exhibited significant differences mainly associated with geographic origin and closure type. Among phenolic families, hydroxycinnamates, phenolic alcohols, and flavonols emerged as the most discriminant compositional domains. Multivariate analysis revealed that wine differentiation was structured by overlapping compositional gradients involving phenolic evolution, color expression, and polysaccharide composition rather than by vintage alone. Overall, the results highlight the multifactorial nature of Chardonnay wine composition and the combined contribution of grape origin, closure-associated bottle evolution, and winemaking factors. Nevertheless, because wines were commercially sourced and bottle age and storage conditions were not standardized, closure-associated differences should be interpreted cautiously as associations rather than causal effects.

## 1. Introduction

Chardonnay (*Vitis vinifera* L.) is one of the most widely cultivated white grape varieties worldwide and represents a key component of the Chilean wine industry in terms of vineyard surface (~10,000 ha) and production volume [1,2]. Its adaptability to diverse climatic conditions and winemaking styles has led to a broad spectrum of wine profiles, ranging from fresh, high-acidity coastal expressions to more structured wines from warmer inland regions. In Chile, this diversity is further enhanced by marked latitudinal and longitudinal gradients, making Chardonnay a suitable model for studying compositional variability in commercial wines.

The phenolic composition of Chardonnay wines is mainly dominated by hydroxycinnamates, which constitute the primary phenolic fraction in white wines [3,4]. In contrast to red wines, flavan-3-ol and tannin concentrations are generally low due to limited skin contact during vinification, resulting in a narrower phenolic range but increased sensitivity to oxidative processes and technological interventions [5,6]. Despite their relatively low concentrations, these compounds play a critical role in color development, oxidative stability, and sensory perception [4,7].

In addition to phenolic compounds, polysaccharides represent key macromolecular constituents of white wines, contributing to mouthfeel, stability, and interactions with other components of the wine matrix [8,9,10]. These compounds originate from both grape cell walls and yeast autolysis, and their composition is strongly influenced by fermentation conditions, lees management, and ageing practices [9,10,11]. Their interaction with phenolic compounds has been shown to modulate astringency perception, viscosity, and overall sensory balance, highlighting their relevance in wine quality beyond basic compositional parameters [8,10,12].

Although several studies have characterized specific aspects of Chardonnay composition, such as phenolic profiles or the effects of winemaking practices [13,14,15], fewer have addressed the integrated behavior of phenolics, polysaccharides, and color attributes in commercial wines. Furthermore, the compositional variability observed in bottled wines is not only driven by grape origin and climatic conditions but also by technological factors, including fermentation management, oxygen exposure, ageing strategies, and closure systems, which can influence wine evolution during storage [4,7,16].

Recent metabolomic studies have demonstrated that the differentiation of Chardonnay wines is better explained by the combined influence of origin and processing history rather than by a single factor such as vintage [17]. In commercial wines, this multicausal variability is particularly relevant, as standardized production practices coexist with diverse vineyard conditions, leading to complex compositional patterns that cannot be fully captured through univariate approaches alone.

Therefore, a comprehensive and multivariate characterization of commercial Chardonnay wines is required to better understand the relative contribution of compositional and technological drivers. In this context, the present study aims to evaluate the physicochemical composition, phenolic profile, polysaccharide fractions, and color attributes of commercial Chardonnay wines from different Chilean regions, considering multiple sources of variability, including geographic origin, vintage, and winemaking-related factors. By integrating univariate and multivariate approaches, this work seeks to provide a holistic interpretation of the chemical diversity of Chardonnay wines and the processes underlying their compositional variability.

## 2. Materials and Methods

### 2.1. Solvents and Chemicals

All reagents and solvents used in this study were of analytical or HPLC grade. Reference standards for the identification and quantification of low-molecular-weight phenolic compounds were selected according to those commonly reported in Chardonnay wines, including gallic acid, protocatechuic acid, caffeic acid, *p*-coumaric acid, caftaric acid, coutaric acid, tyrosol, tryptophol, catechin, epicatechin, *trans*-resveratrol, quercetin, and quercetin-3-*O*-glucoside. *Cis*-resveratrol glucoside (*cis*-piceid) was quantified using the calibration curve of *trans*-resveratrol. All standards, with purity ≥95%, were obtained from commercial suppliers such as Sigma-Aldrich (St. Louis, MO, USA), Extrasynthese (Genay, France), and Phytolab (Vestenbergsgreuth, Germany). Methylcellulose (1500 cP viscosity at 20 g/L), (+)-catechin (purity > 98%), gallic acid (purity > 97%), and malvidin-3-glucoside (purity > 90%) were purchased from Sigma Chemical Co. (St. Louis, MO, USA).

For polysaccharide characterization, nine dextran standards derived from *Leuconostoc mesenteroides*, covering a molecular weight range from 5000 to 670,000 Da, were used to calibrate the gel permeation chromatography columns. Two citrus-derived pectins, present as esterified potassium salts and differing in their degree of esterification (55–70% and 20–34%), were also used as external standards for quantitative analysis. Organic solvents, including methanol, acetonitrile, formic acid, and acetic acid (HPLC grade), as well as ammonium sulphate, vanillin, ethyl acetate, sodium disulfite, diethyl ether, sodium hydroxide, hydrochloric acid, sulfuric acid, and anhydrous sodium sulfate, were supplied by Merck (Darmstadt, Germany). Before chromatographic analysis, all samples were filtered through 0.22 μm polyethylene membrane filters (Millipore, Billerica, MA, USA).

### 2.2. Wine Samples

A total of thirty commercial monovarietal *Vitis vinifera* L. cv. Chardonnay wines corresponding to two consecutive vintages (2023 and 2024) were analyzed. Samples were sourced from major Chilean wine-producing valleys, including Limarí, Casablanca, Leyda, Aconcagua, Colchagua, Maule, and southern regions such as Malleco and Bío-Bío, representing a broad range of climatic conditions and production systems (Table 1, Tables S1 and S2, Figure S1) [18,19,20,21]. In Chile, the influence of the Pacific Ocean plays a key role in shaping vineyard climate, generating cooler coastal zones characterized by lower temperatures and higher humidity, while inland areas, progressively less affected by marine influence, are associated with warmer growing conditions. This climatic gradient provides a relevant framework for interpreting compositional variability among wines from different origins.

All wines were commercially available at the time of sampling and were purchased from specialized wine shops and retailers located in Santiago (Chile) during January and February of 2025, in order to obtain a representative selection of the current market offer. Each sample corresponded to bottled wines produced under standard commercial winemaking conditions, including differences in closure type, and production scale. Table 1 shows the summary of collected samples.

After acquisition, bottles were transported under controlled conditions and stored horizontally in the dark at 15 °C until analysis. All determinations were performed within two months of purchase in order to minimize compositional changes associated with bottle ageing. According to Chilean regulations, wines labeled as varietal must contain at least 85% of the declared cultivar, although Chardonnay wines are generally produced as monovarietal wines in commercial practice [1,2].

### 2.3. Basic Chemical and Spectrophotometric Analyses

Basic oenological parameters, including pH and titratable acidity, were determined according to the official methods established by the International Organisation of Vine and Wine [18]. Titratable acidity was expressed as g H_2_SO_4_·L^−1^. Total phenolic content was estimated by absorbance at 280 nm and expressed as mg gallic acid equivalents (GAE) per liter. Total tannins were determined using the methylcellulose precipitation method adapted for white wines [22]. Color intensity was calculated from absorbance measurements in the visible range using a UV–Vis spectrophotometer (Shimadzu UV-1700, Kyoto, Japan), following the general principles used for wine spectrophotometric characterization [23]. Alcohol content (%vol.) corresponds to those indicated in labeling of each commercial wine. Chilean regulation allows a maximum tolerance (deviation) of 0.5% for alcohol content on wine labels.

### 2.4. HPLC-DAD Analysis of Low-Molecular-Weight Phenolic Compounds

Low-molecular-weight phenolic compounds were analyzed by high-performance liquid chromatography coupled to diode array detector (HPLC–DAD) using an Agilent 1100 Series system (Agilent Technologies, Santa Clara, CA, USA) equipped with a quaternary pump, autosampler, column oven, and diode array detector. Phenolic compounds were extracted by liquid–liquid extraction using diethyl ether and ethyl acetate following previously described procedures [5]. The organic phases were combined, dried over anhydrous sodium sulfate, and evaporated under reduced pressure at 30 °C. The residue was reconstituted in methanol–water (1:1, *v*/*v*) and filtered before injection.

Chromatographic separation was achieved on a reversed-phase C18 column (Nova-Pak, 300 × 3.9 mm, 4 μm; Waters, Milford, MA, USA) at 20 °C. Detection wavelengths were set at 280 nm for phenolic acids and flavanols and 360 nm for flavonols. Compound identification was based on retention times and UV–visible spectra compared with those of authentic standards. Quantification was performed using external calibration curves prepared from individual standards, and for compounds lacking commercial standards, structurally related standards were used. Results were expressed as mg·L^−1^.

### 2.5. Polysaccharide Determination

The molecular-weight distribution of wine polysaccharides was determined by high-performance size exclusion chromatography with refractive index detection (HPSEC–RID), following previously described methodologies [12,14]. Analyses were conducted using an Agilent 1260 Infinity system equipped with a refractive index detector and two Shodex OHpak columns (SB-803 HQ and SB-804 HQ; Showa Denko, Tokyo, Japan) connected in series. Four fractions were considered according to molecular-weight distribution: FI (high molecular weight), FII (medium-molecular weight), FIII (low molecular weight), and FIV (oligosaccharides and peptides). Quantification was performed using calibration curves constructed with dextran and pectin standards. POLYSACCH. accumulated fraction corresponds to the sum of FI, FII, and FIII fractions. The SACCH fraction corresponds to the sum of all fractions (FI, FII, FIII, and FIV). All polysaccharide results are expressed in mg·L^−1^.

### 2.6. Color Measurement (CIELab System)

Color parameters were determined using the CIELab system according to the recommendations of the *Commission Internationale de l’Éclairage* [23,24]. Spectral data were recorded through the measurement of absorbances at 450 nm, 520 nm, 570 nm, and 630 nm using 10 mm path-length quartz cuvettes. The parameters L* (lightness), a* (red–green axis), b* (yellow–blue axis), chroma (C*ab) and hue angle (h*ab) were calculated using the MSCV^®^ software V.7 (2001–2012).

### 2.7. Statistical Analysis

All statistical analyses were performed using GraphPad Prism for Windows 64-bit (version 11.0.0 (84)). Because wine samples did not correspond to identical producers, vineyard origins, or winemaking conditions, the dataset was treated as an unpaired observational design. The explanatory factors considered were vintage, closure type, broad geographic location, and valley of origin, as previously stated in Table 1. For the factors accounting for two groups (vintage, closure, and broad geographic location), unpaired Welch’s *t*-test was applied to assess statistical differences. When datasets did not meet normal distribution (Shapiro–Wilk (W) test), logarithmic normalization was applied to the data. If normalization was not possible, the non-parametric Mann–Whitney test was employed instead. For the factor of valley of origin, an unpaired ordinary one-way ANOVA followed by Tukey’s multiple comparisons test were employed when datasets met the assumptions of normality and homoscedasticity. For non-normal datasets, logarithmic normalization was applied, and when normalization was not possible, the non-parametric Kruskal–Wallis test was used. The datasets that did not pass the Brown–Forsythe test for equality of variances were treated following the Brown–Forsythe and Welch ANOVA tests. Statistical significance was established at α = 0.05 in all cases.

Multivariate analysis was performed by principal component analysis (PCA) using standardized variables (scale data to have a mean of 0 and SD of 1), and principal components were selected based on eigenvalues following the Kaiser rule. PCA was used to visualize global compositional variability and clustering patterns among wines.

Climatic and bioclimatic contextualization of the vineyard regions was based on official Chilean reports and agroclimatic datasets [18,19,20,21]. Correlation analysis among low-molecular-weight phenolics, polysaccharide fractions, and color variables was conducted using Spearman r coefficients, and heatmap of such correlations are shown in the Supplementary Materials (Figure S2).

## 3. Results and Discussion

The basic oenological parameters of the commercial Chardonnay wines analyzed are presented in Supplementary Table S3. Overall, alcohol content, pH, titratable acidity, and residual sugar showed relatively low variability among samples, reflecting the strong technological standardization commonly observed in commercial white winemaking. This compositional homogeneity in conventional oenological parameters contrasts with the greater variability observed in phenolic composition, polysaccharide fractions, and color-related parameters.

The significance levels obtained for all studied variables according to vintage, closure type, broad geographic location, and valley of origin are summarized in Table 2. As shown in this table, only a subset of the analyzed variables exhibited significant differences for at least one of the tested factors. These significant cases, highlighted in gray in Table 2, correspond to the parameters that will be specifically addressed and discussed in the following sections. Overall, the results indicate that the response of the Chardonnay wine matrix was not uniform across analytical domains, and that the compositional variability of the wines is better interpreted as a multifactorial phenomenon rather than as a simple vintage effect.

Accordingly, the Results and Discussion section focuses only on those variables for which statistically significant differences were detected in Table 2, emphasizing their chemical interpretation and their relevance for understanding the diversity of commercial Chilean Chardonnay wines.

Basic oenological parameters and total phenols showed limited discriminatory power, whereas significant effects were more frequently observed for total tannins, selected CIELab color coordinates, several polysaccharide fractions, and several low-molecular-mass phenolic compounds and grouped phenolic families. Importantly, these significant responses were not associated with a single explanatory factor, but rather with different combinations of vintage, closure type, geographic location, and valley of origin.

### 3.1. Total Tannins and Color-Related Variables

**Figure 1:**
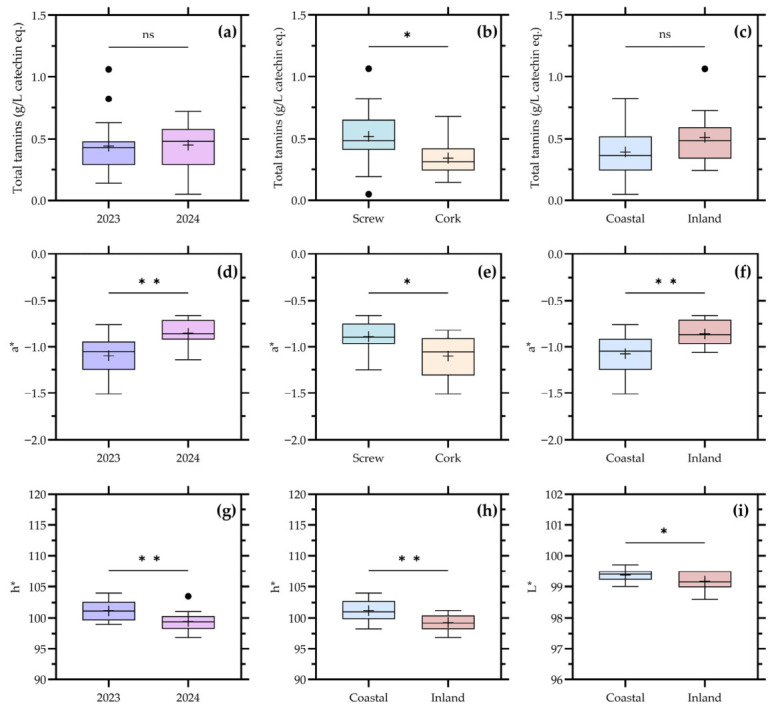
Distribution of total tannins and CIELab color parameters in commercial Chardonnay wines according to vintage, closure type, and location. Boxplots show total tannins (**a**–**c**) and CIELab color coordinates (**d**–**i**) for the factors in which significant differences were detected. Boxes represent the interquartile range, the central line indicates the median, whiskers indicate data dispersion, and (+) indicates mean values. Statistical significance is indicated as follows: ns, not significant; * *p* < 0.05; ** *p* < 0.01.

The distribution of total tannins and CIELab color coordinates according to the significant factors identified in Table 2 are presented in Figure 1.

Total tannins showed significant differences as a function of closure type and valley of origin, whereas no significant effect of vintage or location was observed. Wines sealed with cork and screw cap exhibited distinct tannin distributions, indicating that post-bottling conditions contributed to differences in the measurable tannin fraction. In addition, the valley factor revealed a clear differentiation, suggesting that regional origin influenced tannin levels despite the overall low concentrations expected for white wines.

Regarding color parameters, a* was significantly affected by vintage, closure, and location, while hue angle (h*) showed significant differences for vintage, location, and valley. Lightness (L*) was influenced by location, whereas b* and chroma (C*) did not reach statistical significance, although some tendencies were observed. In particular, wines from different broad geographic locations (coastal vs. inland) showed distinct distributions for a* and h*, indicating that both the green–red axis and overall hue were sensitive to geographic origin.

From a compositional standpoint, these results highlight a clear contrast between global phenolic indicators and more specific or derived variables. Total phenols did not show significant differences across any factor (Table 2), whereas total tannins responded significantly to closure and valley. This suggests that, in the studied wines, the tannin fraction is more sensitive to both origin and post-bottling evolution than the bulk phenolic pool, which is largely dominated by hydroxycinnamates [3,4,22].

The association between closure type and total tannins is particularly noteworthy. Oxygen transfer through the closure is known to influence the oxidative evolution of wine phenolics, even in white wines where tannin levels are relatively low. Differences in oxygen transmission associated with closure systems may contribute to differential polymerization, precipitation, or degradation pathways affecting condensed phenolics during bottle storage, leading to measurable differences in tannin content over time [7,22]. This interpretation is consistent with the observed separation between closure groups in Figure 1.

The significant influence of valley on total tannins further indicates that grape origin remains an important determinant of phenolic extraction potential, even under commercial winemaking conditions that typically limit skin and seed contact. Regional differences in grape composition, including seed maturity and phenolic profile, as well as variations in pressing intensity and clarification practices, may contribute to this variability [5,16].

Color-related variables showed a more complex and multifactorial response. The sensitivity of a* and hue angle (h*) to vintage, location, and, in the case of h*, valley, indicates that chromatic expression in Chardonnay wines is not solely dependent on phenolic concentration but also on the transformation and oxidation state of phenolic compounds. In white wines, small changes in hydroxycinnamates and their oxidation products can lead to perceptible differences in color, particularly along the green–yellow–brown axis [7,22].

The significant effect of location on a*, L*, and h* suggests that environmental conditions associated with coastal versus inland regions influenced either the initial phenolic composition of the grapes or the subsequent evolution of the wines. Differences in solar radiation, temperature amplitude, and grape exposure may affect flavonol content and hydroxycinnamate levels, which in turn modulate color development [1,22]. Moreover, the additional effect of closure on a* supports the idea that post-bottling oxygen availability contributes to the evolution of color attributes.

Overall, the results presented in Figure 1 demonstrate that, in commercial Chardonnay wines, color parameters and total tannins provide a higher resolution for detecting compositional differences than total phenols. These variables integrate both grape-derived characteristics and technological factors, including closure type and oxidative evolution, making them particularly informative for understanding variability in wine appearance and structure.

### 3.2. Polysaccharide Fractions and Total Polysaccharides

**Figure 2:**
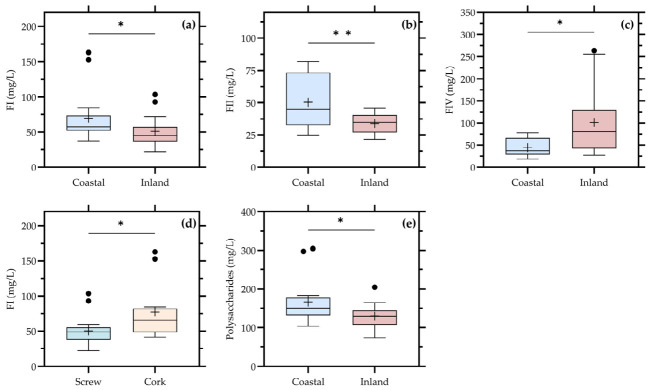
Distribution of polysaccharide fractions and total polysaccharides in commercial Chardonnay wines according to closure type, location, and valley of origin. Boxplots represent the molecular-weight fractions FI–FIV (**a**–**d**) and the accumulated polysaccharide fraction (FI + FII + FIII) (**e**) for the factors in which significant differences were detected. Boxes represent the interquartile range, the central line indicates the median, whiskers indicate data dispersion, and (+) indicates mean values. Statistical significance is indicated as follows: * *p* < 0.05; ** *p* < 0.01.

The distribution of polysaccharide fractions according to molecular weight and their pooled concentration is shown in Figure 2.

According to the significance pattern summarized in Table 2, polysaccharide composition was not driven by vintage, but instead showed significant differences mainly associated with closure type, geographic location, and valley of origin. Specifically, FI was significantly affected by closure and location, FII by location and especially by valley, and FIV by both location and valley. In addition, the pooled polysaccharide fraction (POLYSACH, corresponding to the accumulated concentrations of FI, FII, and FIII) also showed significant differences for location and valley, whereas FIII remained non-significant across all factors.

These results indicate that the variability of polysaccharides in commercial Chardonnay wines is not primarily related to inter-annual climatic conditions, but rather to origin and technological factors. This is consistent with the well-established understanding that wine polysaccharides originate from both grape cell walls and yeast metabolism, particularly through the release of mannoproteins during fermentation and lees ageing [8,9,10,11,25,26]. Consequently, their final concentration and molecular-weight distribution reflect the combined influence of grape composition, extraction conditions, and post-fermentative processes.

The significant effect of closure on FI is particularly relevant. High-molecular-weight polysaccharides are closely associated with colloidal stability and mouthfeel properties, and their behavior may be influenced by oxidative conditions during bottle ageing.

Differences associated with oxygen transmission properties of closure systems may contribute to variations in the degradation, aggregation, or persistence of these macromolecules during bottle evolution, thereby contributing to the observed differences [7,22]. This suggests that closure type not only impacts phenolic evolution but also the structural components of the wine matrix.

The strong influence of location and valley on FII, FIV, and total polysaccharides highlights the importance of origin-related factors. Geographic origin integrates multiple variables, including climate, soil properties, and typical winemaking practices. Grape maturity at harvest may further influence this variability through changes in berry cell wall composition, pectin solubilization, and the extractability of grape-derived polysaccharides during pressing. Although skin contact is limited in white winemaking compared with red winemaking, differences in ripening stage and pressing conditions may still affect the transfer of soluble cell-wall polysaccharides into Chardonnay musts and wines [9,11]. Differences in pressing intensity, juice clarification, fermentation conditions, and lees management among regions and wineries may lead to distinct extraction and release patterns of polysaccharides [6,8,10]. In particular, the high sensitivity of FII and FIV to valley suggests that medium-molecular-weight and oligosaccharide fractions are especially responsive to regional production styles and processing conditions.

In addition to geographic origin, part of the observed variability in polysaccharide composition may also reflect differences in commercial winemaking practices among producers. Lees ageing duration, fermentation management, clarification intensity, and barrel maturation can substantially influence the release and persistence of mannoproteins and other soluble polysaccharides in Chardonnay wines [8,9,10,14]. In particular, ageing on lees has been associated with increased extraction of yeast-derived polysaccharides and modifications in wine matrix interactions, potentially contributing to differences in colloidal stability and mouthfeel properties [14]. Therefore, the polysaccharide variability observed in the present study likely reflects the combined contribution of regional origin and post-fermentative technological decisions.

By contrast, the absence of significant differences for FIII suggests that this fraction may represent a more conserved component of the Chardonnay polysaccharide matrix, less affected by either origin or technological variability. This behavior has been previously reported for certain intermediate molecular-weight fractions, which may exhibit lower sensitivity to both extraction and degradation processes [8,10].

From a functional perspective, these findings are highly relevant. Polysaccharides, especially mannoproteins and arabinogalactans, are known to play a key role in modulating mouthfeel attributes such as viscosity, smoothness, and balance, as well as in influencing interactions with phenolic compounds and aroma molecules [6,7,8,9,10]. The fact that these fractions are more strongly associated with location and valley than with vintage suggests that textural differences among commercial Chardonnay wines are largely determined by regional style and winemaking practices rather than by seasonal variation.

From a sensory perspective, these compositional differences may be particularly relevant for the perception of texture and palate integration in Chardonnay wines. Previous studies have shown that wine polysaccharides, especially mannoproteins and arabinogalactans, may modulate viscosity, smoothness, palate hotness, and overall mouthfeel balance in white wines [8,9,10]. In addition, interactions between polysaccharides and phenolic compounds may influence the perception of bitterness and tactile properties, contributing to stylistic differences among commercial Chardonnay wines. Therefore, the variability observed in polysaccharide fractions could potentially have important sensory implications associated with regional style and ageing practices.

Overall, the results presented in Figure 2 reinforce the concept that polysaccharide composition in commercial wines should be interpreted as a marker of both origin and processing history. Unlike basic oenological parameters, which remain relatively stable, and unlike some phenolic compounds, which may show selective vintage effects, polysaccharides integrate multiple stages of wine production and evolution, providing valuable insight into the structural and sensory complexity of Chardonnay wines.

### 3.3. Valley-Related Differences in Oenological, Color, and Polysaccharide Parameters

**Figure 3:**
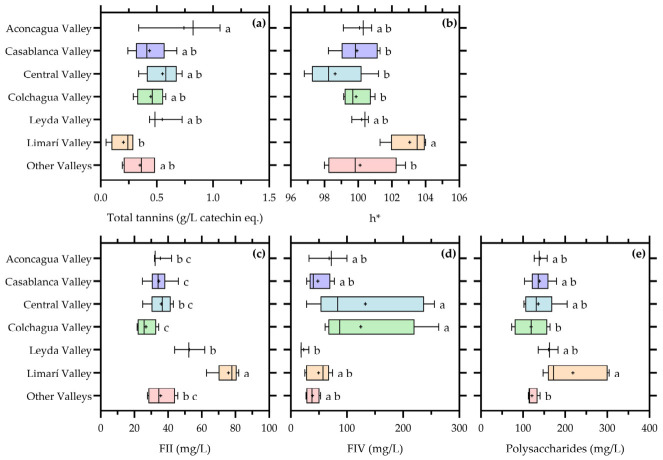
Valley-related differences in (**a**) Total tannins; (**b**) CIELab h* coordinate; (**c**) fraction II of polysaccharides; (**d**) fraction IV of polysaccharides; (**e**) Summatory of fractions I, II and III of polysaccharides in commercial Chardonnay wines. Boxplots show the variables for which significant differences among valleys were detected. Boxes represent the interquartile range, the central line indicates the median, whiskers indicate data dispersion, and (+) indicates mean values. Different letters indicate significant differences among valleys according to the corresponding multiple-comparison test (*p* < 0.05).

The effect of valley of origin on the main compositional variables showing statistical significance is summarized in Figure 3.

As previously indicated in Table 2, basic oenological parameters such as alcohol content, pH, and titratable acidity did not show significant differences among valleys. This reinforces the idea that these parameters are strongly regulated during winemaking and therefore exhibit limited sensitivity to geographic origin in commercial wines [16,17,27].

In contrast, clear valley-dependent patterns emerged for several color and structural variables, shown in Figure 3. Hue angle (h*) showed significant differences among valleys, indicating that chromatic expression varied according to origin. This suggests that regional factors influenced the balance between yellow, green, and potentially oxidative tonalities. Given that hue in white wines is strongly linked to the oxidation state of hydroxycinnamic derivatives and related compounds, these differences likely reflect variations in both grape composition and oxidative evolution pathways [7,22].

Similarly, total tannins also exhibited significant differences among valleys, confirming that regional origin influences the extraction and/or retention of this fraction, even in white wines. Although tannin concentrations are generally low in Chardonnay, differences in grape maturity, seed composition, and pressing conditions may lead to measurable variability among regions [5,16]. These results are consistent with the interpretation that tannins, unlike total phenols, retain some sensitivity to origin even under commercial production conditions.

Polysaccharide fractions showed some of the most pronounced valley effects. In particular, FII, FIV, and the pooled polysaccharide fraction displayed significant differences among valleys, as evidenced by the separation of groups indicated by different letters in Figure 3. This confirms that the molecular-weight distribution of polysaccharides is strongly influenced by regional factors. These may include differences in grape cell wall composition, as well as variations in fermentation management, yeast autolysis, and lees contact, which are often characteristic of specific production areas [6,8,9,10].

The marked differentiation observed for FII and FIV suggests that medium-molecular-weight polysaccharides and oligosaccharides are particularly sensitive to regional winemaking styles and processing conditions. These fractions are known to be associated with yeast-derived mannoproteins and degradation products of higher molecular-weight polysaccharides, which can vary depending on fermentation kinetics and ageing practices [8,10,25,26]. Therefore, their variability among valleys likely reflects differences in both grape origin and cellar practices.

Furthermore, differences in regional winemaking approaches may also contribute to the observed valley-dependent polysaccharide patterns. Commercial Chardonnay production frequently incorporates practices such as barrel fermentation or ageing on lees, both of which promote yeast autolysis and the release of soluble polysaccharides into the wine matrix [14]. Consequently, part of the regional differentiation observed for FII and FIV may reflect not only grape origin but also differences in maturation strategies among wineries.

Overall, Figure 3 highlights that valley of origin constitutes a meaningful source of compositional variability in commercial Chardonnay wines, even within a heterogeneous dataset. Importantly, this variability is not expressed through basic oenological parameters, but rather through more sensitive indicators such as color attributes, tannins, and polysaccharide fractions. This supports the concept that regional identity in Chardonnay wines is primarily conveyed through oxidation-sensitive compounds and matrix-related components, rather than through global physicochemical variables.

These findings reinforce the need to consider valley not only as a geographic descriptor, but as an integrative factor encompassing climate, vineyard conditions, and typical winemaking approaches. In this context, the compositional differences observed among valleys provide valuable insight into how regional factors contribute to the structural and sensory diversity of commercial Chardonnay wines.

### 3.4. Low-Molecular-Mass Phenolics: Individual Compounds

**Figure 4:**
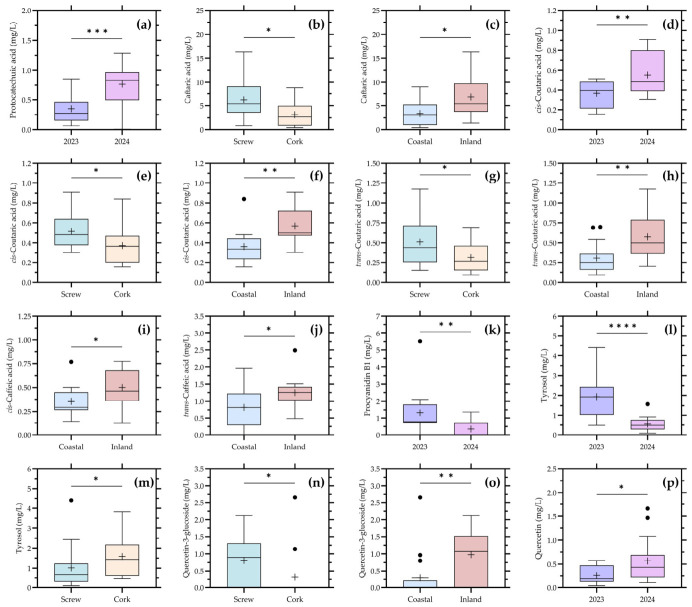
Distribution of individual low-molecular-mass phenolic compounds in commercial Chardonnay wines according to vintage (**a**,**d**,**k**,**l**,**p**), closure type (**b**,**e**,**g**,**m**,**n**), and location (**c**,**f**,**h**,**i**,**j**,**o**). (**a**) Protocataechuic acid; (**b**) Caftaric acid; (**c**) Caftaric acid; (**d**) *cis*-Coutaric acid; (**e**) *cis*-Coutaric acid; (**f**) *cis*-Coutaric acid; (**g**) *trans*-Coutaric acid; (**h**) *trans*-Coutaric acid; (**i**) *cis*-Caffeic acid; (**j**) *trans*-Caffeic acid; (**k**) Procyanidin B1; (**l**) Tyrosol; (**m**) Tyrosol; (**n**) Quercetin-3-glucoside; (**o**) Quercetin-3-glucoside; (**p**) Quercetin aglycone. Boxplots represent the compounds for which significant differences were detected for at least one explanatory factor. Boxes represent the interquartile range, the central line indicates the median, whiskers indicate data dispersion, and (+) indicates mean values. Statistical significance is indicated as follows: * *p* < 0.05; ** *p* < 0.01; *** *p* < 0.001; **** *p* < 0.0001.

The distribution of individual low-molecular-mass phenolic compounds according to the significant factors identified in Table 2 is presented in Figure 4.

Overall, the results reveal a heterogeneous response depending on the phenolic family considered. Unlike global parameters such as total phenols, which did not show significant differences, several individual compounds exhibited statistically significant effects associated with vintage, closure type, location, and valley, confirming that low-molecular-mass phenolics provide a higher resolution for detecting compositional variability.

Among hydroxybenzoic compounds, protocatechuic acid showed a highly significant vintage effect, together with a significant valley effect. In addition, the grouped benzoic acids also differed significantly according to vintage and valley. This suggests that this phenolic family retains some sensitivity to inter-annual conditions, while also reflecting regional differentiation. Given that benzoic derivatives may originate from oxidative degradation pathways of hydroxycinnamic precursors, their behavior is consistent with both climatic and process-related influences [3,4,7,22].

Hydroxycinnamates showed one of the most complex and informative patterns. Caftaric acid differed significantly according to closure, location, and valley, whereas *cis*-coutaric acid was significant for all four factors (vintage, closure, location, and valley). *Trans*-coutaric acid also showed significant differences for closure and location. In addition, grouped hydroxycinnamates were significantly influenced by closure and location. These results clearly indicate that hydroxycinnamate chemistry in commercial Chardonnay wines is strongly modulated by both origin and technological factors, particularly those related to oxygen-mediated evolution. This is consistent with the central role of hydroxycinnamic acids in oxidative reactions and browning processes in white wines [7,22].

Hydroxycinnamates such as caftaric and coutaric acids are among the principal oxidation-sensitive phenolics in white wines and play a major role in browning development during bottle ageing [7]. Their oxidation leads to the formation of quinones and secondary reaction products that can modify both chromatic coordinates and sensory perception. Consequently, the significant differences observed among closure types and geographic locations likely reflect differences in oxidative evolution pathways occurring before and after bottling.

The fact that closure type significantly affected several hydroxycinnamates supports the hypothesis that post-bottling oxygen availability plays a key role in shaping their evolution. Differences in oxygen transmission between cork and screw cap closures can lead to distinct oxidation trajectories, influencing both the degradation and transformation of these compounds [7,19]. At the same time, the strong effect of location suggests that grape composition and initial phenolic profiles also contribute to the observed variability.

In addition, differences in grape maturity at harvest among regions and producers may also have contributed to the variability observed for several phenolic compounds. Ripening stage is known to influence the accumulation and extractability of hydroxycinnamic acids, flavonols, and seed-derived phenolics in white grapes, while harvest decisions may additionally affect oxidation susceptibility and subsequent wine evolution [5,17,22]. Because the wines analyzed in this study were commercially produced, harvest timing and maturity indices were not standardized, and therefore these factors should also be considered as potential contributors to the compositional variability observed across the dataset.

Within flavanols, only procyanidin B1 showed a significant vintage effect, whereas catechin, epicatechin, and the grouped flavanol fraction remained non-significant. This indicates that, in Chardonnay wines, the flavanol pool is generally stable and of limited variability, as expected due to restricted extraction from skins and seeds during white winemaking [5,16]. However, the sensitivity of procyanidin B1 to vintage suggests that specific dimeric forms may still reflect differences in grape maturity or extraction conditions between years.

Yeast-derived phenolic alcohols displayed a strong response to multiple factors. Tyrosol showed a highly significant vintage effect, as well as a significant closure effect, while the grouped phenolic alcohol fraction was influenced by vintage, closure, and location. These results highlight the dual origin of these compounds: although they are primarily formed during fermentation through yeast metabolism, their final concentration may also be affected by grape composition and post-fermentative evolution [17]. The sensitivity of yeast derived phenolic alcohols (tyrosol and tryptophol) to closure type further suggests that bottle ageing conditions may influence their stability or transformation.

Flavonols emerged as another highly discriminant group. Quercetin-3-glucoside showed significant differences for closure, location, and valley, with a near-significant effect for vintage, while quercetin was significantly affected by vintage. The grouped flavonol fraction also showed significant effects for vintage and location. These findings are particularly relevant because flavonols are known to be strongly influenced by grape exposure to light and UV radiation and, thus, are closely linked to vineyard conditions [17,22]. Their sensitivity to both location and closure suggests that they integrate vineyard-derived signals with subsequent oxidative and hydrolytic transformations during winemaking and bottle ageing.

By contrast, stilbenes did not show significant differences for any factor, indicating that these compounds had limited variability and low discriminant power within the present dataset. This is consistent with their typically low concentrations in white wines and their dependence on specific stress conditions or extraction processes [5,22].

Overall, the results presented in Figure 4 demonstrate that low-molecular-mass phenolics in commercial Chardonnay wines are structured by a combination of factors rather than by a single dominant variable. Hydroxycinnamates, phenolic alcohols, and flavonols appear as the most responsive and informative chemical families, reflecting the combined influence of grape origin, environmental conditions, and technological practices, including closure-related bottle evolution. These findings reinforce the need to interpret phenolic composition within a multifactorial framework that integrates both vineyard and winemaking effects.

### 3.5. Low-Molecular-Mass Phenolics Grouped by Chemical Family

**Figure 5:**
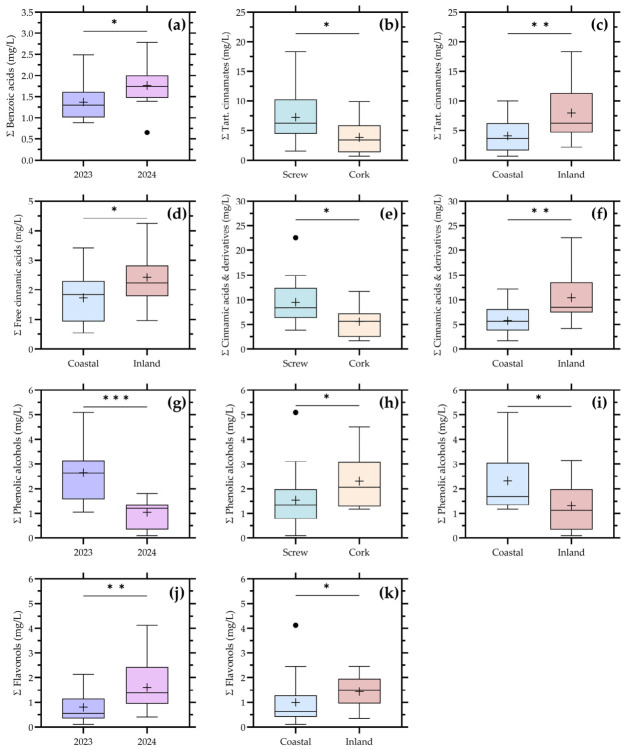
Distribution of low-molecular-mass phenolic compounds grouped by chemical family. (**a**) Benzoic acids; (**b**) Tartaric acid ester cinnamates; (**c**) Tartaric acid ester cinnamates; (**d**) Cinnamic acids; (**e**) Cinnamic acids and derivatives; (**f**) Cinnamic acids and derivatives; (**g**) Yeast phenolic alcohols; (**h**) Yeast phenolic alcohols; (**i**) Yeast phenolic alcohols; (**j**) Flavonols; and (**k**) Flavonols according to vintage (**a**,**g**,**j**), closure type (**b**,**e**,**h**), and location (**c**,**d**,**f**,**i**,**k**). Boxplots represent grouped concentrations of benzoic acids, several hydroxycinnamates, flavanols, phenolic alcohols, stilbenes, and flavonols for the factors in which significant differences were detected. Boxes represent the interquartile range, the central line indicates the median, whiskers indicate data dispersion, and (+) indicates mean values. Statistical significance is indicated as follows: * *p* < 0.05; ** *p* < 0.01; *** *p* < 0.001.

A complementary view of phenolic variability is provided in Figure 5, where individual compounds are integrated into broader chemical families, facilitating the interpretation of the main compositional drivers in commercial Chardonnay wines.

According to Table 2, differentiated patterns emerged among phenolic families. Benzoic acids showed significant effects for both vintage and valley, indicating that this group retains sensitivity to inter-annual variability while also reflecting regional differentiation. This behavior is consistent with their formation through oxidative degradation pathways of hydroxycinnamic precursors, which are influenced by both grape composition and post-fermentative evolution [3,4,7,22]. Moreover, previous studies have shown that benzoic derivatives may increase or fluctuate during bottle ageing as a consequence of phenolic transformation reactions, including oxidation and hydrolysis [28].

Hydroxycinnamate fractions, including tartaric esters and their derivatives, were significantly affected by closure type and geographic location, whereas free hydroxycinnamic acids were mainly influenced by location. This confirms that this phenolic domain is particularly sensitive to both grape-derived factors and oxygen-mediated processes. Hydroxycinnamic acids are key substrates in oxidative reactions and browning pathways in white wines, and their evolution is strongly modulated by oxygen availability during storage [7,22]. The significance of closure type observed in this study is therefore consistent with previous reports demonstrating that different stoppers regulate oxygen ingress and consequently affect phenolic evolution and color development in bottled Chardonnay wines. In addition, experimental evidence has shown that phenolic compounds, particularly hydroxycinnamates and flavan-3-ols, tend to decrease during bottle ageing due to oxidation and polymerization reactions, further supporting the patterns observed here.

Phenolic alcohols exhibited one of the strongest multifactorial responses, being significantly affected by vintage, closure, and location. This confirms that this group integrates both fermentation-derived and post-bottling influences. Compounds such as tyrosol are primarily produced during fermentation but can be further modulated during ageing through oxidative and metabolic processes [17]. The additional effect of closure supports the role of bottle evolution in shaping their final concentration, in line with studies showing that storage conditions and oxidative evolution exposure influence the evolution of phenolic-related metabolites in white wines [28,29].

Flavonols also showed significant differences, particularly for vintage and location. These compounds are closely associated with grape exposure to solar radiation and vineyard conditions, but they are also susceptible to oxidative and hydrolytic transformations during wine ageing [17,22]. Their persistence as discriminant variables suggests that they retain a partial vineyard-derived signal while also integrating post-fermentative evolution, a behavior consistent with previous metabolomic studies in Chardonnay wines [1].

By contrast, flavanols and stilbenes did not show significant differences when considered as grouped families. This limited discriminatory power is expected, as flavanols are typically present at low concentrations in white wines due to restricted extraction, while stilbenes are minor components that depend on specific stress-related pathways [5,16,22]. Furthermore, several studies have shown that many low-molecular-weight phenolics tend to decrease markedly during bottle ageing, which may reduce their variability at the commercial scale.

Overall, the grouped-family analysis reinforces that phenolic variability in commercial Chardonnay wines is not driven by a single factor, but rather by the interaction between grape origin, technological practices, and bottle evolution. In particular, hydroxycinnamates, phenolic alcohols, and flavonols emerge as the most informative phenolic domains, as they integrate both environmental and process-related influences.

In addition to vineyard-related factors, part of the variability observed among phenolic families may also derive from technological interventions commonly applied during commercial white winemaking. Clarification treatments such as bentonite fining can reduce hydroxycinnamic acids and flavanol concentrations, while fermentation conditions, lees management, and ageing strategies may further modulate phenolic evolution and matrix interactions [14,15]. Therefore, the phenolic differences observed among wines likely reflect the combined influence of grape origin and winery-dependent processing decisions.

### 3.6. Valley Effect on Low-Molecular-Mass Phenolics

**Figure 6:**
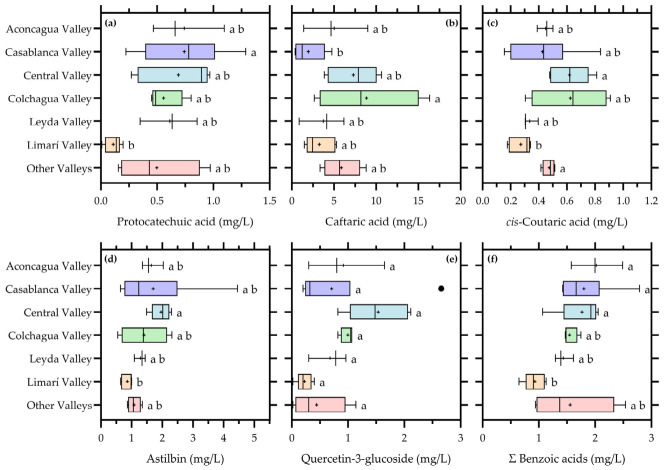
Valley-related differences in low-molecular-mass phenolic compounds in commercial Chardonnay wines. (**a**) Protocatechuic acid; (**b**) Caftaric acid; (**c**) *cis*-Coutaric acid; (**d**) Astilbin; (**e**) Quercetin-3-glucoside; (**f**) Summatory of benzoic acids. Boxplots show the individual compounds and grouped phenolic families for which significant differences among valleys were detected. Boxes represent the interquartile range, the central line indicates the median, whiskers indicate data dispersion, and (+) indicates mean values. Different letters indicate significant differences among valleys according to the corresponding multiple-comparison test (*p* < 0.05).

The influence of valley of origin on low-molecular-mass phenolics is detailed in Figure 6, confirming that geographic origin remains a significant source of compositional variability.

Hydroxycinnamates, particularly caftaric acid and *cis*-coutaric acid, showed clear differentiation among valleys. These compounds are highly sensitive to both grape composition and oxidative processes, and their variability suggests that regional differences in grape maturity, vineyard conditions, and grape juice handling contributed to the observed patterns. In addition, their previously demonstrated sensitivity to oxygen exposure reinforces the idea that valley-related differences may also reflect distinct winemaking and storage practices [7,22,29].

Protocatechuic acid and grouped benzoic acids also displayed significant valley effects, supporting the interpretation that oxidative degradation pathways vary among regions. As reported in bottle ageing studies, the transformation of hydroxycinnamic acids into benzoic derivatives is influenced by both oxygen availability and storage time, indicating that regional differences may extend beyond grape composition to include differential oxidative trajectories.

Flavonols, particularly quercetin-3-glucoside and astilbin, showed significant differentiation among valleys. These compounds are strongly associated with vineyard conditions such as solar radiation, canopy structure, and climatic factors [17,22]. Their persistence in bottled wines indicates that flavonols retain a robust vineyard-derived signature, even after fermentation and ageing processes.

Importantly, not all phenolic compounds showed significant valley effects, highlighting the selective nature of geographic influence. Compounds involved in oxidation pathways or linked to grape exposure were more responsive, whereas others remained relatively stable or were more strongly governed by technological factors. This reinforces the concept that regional identity in Chardonnay wines is expressed primarily through oxidation-sensitive and vineyard-dependent phenolic fractions.

### 3.7. Multivariate Analysis of Compositional Variability (PCA)

**Figure 7:**
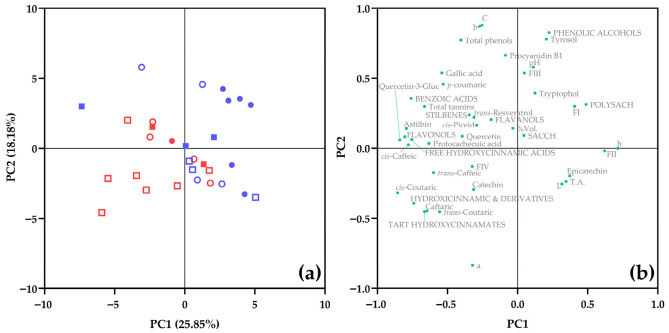
Principal component analysis of compositional variability in commercial Chardonnay wines. (**a**) Score plot showing the distribution of samples according to the first two principal components. Circles (●/○) correspond to 2023 wines and squares (■/□) to 2024 wines. Filled symbols (●/■) indicate cork closure, and open symbols (○/□) indicate screw cap. Blue symbols (●/○/■/□) represent coastal wines and red symbols (●/○/■/□) represent inland wines. (**b**) Loading plot showing the contribution of the analytical variables included in the model. PCA was performed using standardized variables.

The PCA results (Figure 7) provide an integrated representation of the compositional variability, revealing substantial overlap among samples and indicating that no single factor alone accounts for the observed chemical differentiation.

Vintage did not drive a clear separation of samples, in agreement with the univariate analyses. Instead, a more structured organization emerged when closure type and geographic location were jointly considered. The partial grouping of coastal and inland wines suggests that broad environmental conditions contribute to shaping the phenolic matrix and associated compositional features, although not in a deterministic manner [1,22].

Closure-associated bottle evolution may have contributed to the sample distribution patterns observed in the PCA. This observation aligns with the established role of closure systems in controlling oxygen ingress and modulating wine evolution during bottle storage. Previous studies have shown that differences in oxygen transmission properties among closures may affect oxidation kinetics and wine evolution during bottle storage, leading to divergent trajectories in phenolic transformation, color development, and overall chemical stability [7,22,29]. In this context, the contribution of closure to the PCA structure likely reflects cumulative oxidative processes rather than immediate compositional differences at bottling.

In parallel, the observed gradients are consistent with the progressive modification of phenolic compounds during ageing. Oxidation and polymerization reactions can lead to a general decrease in reactive phenolics, accompanied by changes in chromatic properties and sensory attributes [28]. These processes are particularly relevant for hydroxycinnamates, which play a central role in white wine oxidation chemistry, and whose evolution depends strongly on oxygen availability and matrix conditions [9,22].

Taken together, the PCA supports a process-oriented interpretation of Chardonnay wine composition, consistent with the multifactorial conceptual framework proposed in Supplementary Figure S3. Rather than reflecting discrete categorical differences, the data describe a compositional continuum shaped by the interaction among grape origin, environmental conditions, technological modulation, and closure-associated bottle evolution. The absence of clear clustering by vintage, combined with the partial structuring associated with closure and location, underscores the hierarchical and multifactorial nature of compositional variability in commercial Chardonnay wines [6,7,22].

## 4. Conclusions

The present study demonstrates that the chemical variability of Chilean commercial Chardonnay wines cannot be adequately explained by vintage alone. Instead, compositional differences arise from the combined and hierarchical influence of closure type, broad geographic location, and valley of origin, which frequently exert effects comparable to or greater than those associated with year.

Basic oenological parameters and total phenols showed limited discriminatory power, confirming their strong technological regulation in commercial wines. In contrast, total tannins, selected CIELab coordinates, specific polysaccharide fractions, and several low-molecular-mass phenolics were significantly modulated by closure, location, and/or valley. Among phenolic compounds, hydroxycinnamates, phenolic alcohols, and flavonols emerged as the most informative chemical domains, whereas grouped flavanols and stilbenes contributed less to sample differentiation. Polysaccharides were primarily influenced by geographic origin, reinforcing their role as integrative markers of grape composition and post-fermentative processes.

Multivariate analysis further confirmed that wine composition is structured along overlapping gradients rather than discrete groupings, reflecting the interaction between grape-derived factors and technological modulation, including closure-associated bottle evolution and oxidative trajectories during bottle ageing.

One limitation of the present study is the absence of sensory evaluation, which restricts the direct interpretation of how the observed compositional differences may translate into perceptible sensory properties or wine quality perception. Future studies integrating sensory analysis with phenolic, polysaccharide, and color characterization would provide a more comprehensive understanding of Chardonnay wine typicity, mouthfeel, and regional style differentiation.

Furthermore, bottle age and storage history prior to acquisition were not standardized because the wines were commercially sourced, and therefore closure-associated compositional differences should be interpreted cautiously as part of broader bottle-evolution trajectories rather than as direct closure effects.

Overall, these findings support a shift from a vintage-centered interpretation toward a multifactorial framework in which commercial Chardonnay wines are best understood as process-modulated matrices under heterogeneous commercial production and bottle-storage conditions. This perspective provides a more realistic basis for interpreting compositional variability and highlights the importance of integrating origin and technological factors when assessing wine style, quality, and typicity in modern commercial systems.

## Figures and Tables

**Table 1:** Summary of commercial Chardonnay wines analyzed.

Factor	Groups	
Vintage	2023 (n = 15)	2024 (n = 15)
Closure type	Cork (n= 12)	Screw cap (n = 18)
Location	Coastal (n = 16)	Inland (n = 14)
Valley	Aconcagua Valley (n = 3)	
	Casablanca Valley (n = 6)	
	Central Valley (n = 5)	
	Colchagua Valley (n = 4)	
	Leyda Valley (n = 3)	
	Limarí Valley (n = 5)	
	Other ^a^ (n = 4)	

^a^ Other Valleys include San Antonio Valley, Cachapoal Valley, Bío-Bío Valley, and Malleco Valley.

**Table 2:** *p*-values of each studied parameter employing as a factor the Vintage (2023 vs. 2024), Closure (Cork vs. Screw cap), Location (Coastal vs. Inland), and Valley (Origin Denomination producing areas).

Parameter	Vintage Factor	Closure Factor	Location Factor	Valley Factor
%Vol.	0.6773 ^a^	0.5863 ^b^	0.9861 ^b^	0.5788 ^d^
pH	0.3817 ^a^	0.1711 ^a^	0.0651 ^c^	0.1279 ^e^
T.A. (H_2_SO_4_ eq.)	0.7525 ^a^	0.1407 ^c^	0.9548 ^a^	0.7442 ^e^
Total phenols	0.2779 ^a^	0.2705 ^a^	0.4670 ^a^	0.2043 ^e^
Total tannins	0.9280 ^a^	0.0156 ^a^	0.1388 ^a^	0.0088 ^e^
a*	0.0011 ^a^	0.0117 ^a^	0.0039 ^a^	0.1700 ^e^
b*	0.3078 ^a^	0.0614 ^a^	0.6319 ^a^	0.1814 ^e^
L*	0.7356 ^a^	0.9770 ^a^	0.0440 ^a^	0.4298 ^e^
C*	0.2694 ^a^	0.0538 ^a^	0.5720 ^a^	0.1953 ^e^
h*	0.0072 ^a^	0.3055 ^a^	0.0017 ^a^	0.0018 ^e^
POLYSACCH FI	0.1838 ^b^	0.0176 ^c^	0.0269 ^b^	0.2789 ^e^
POLYSACCH FII	0.0603 ^c^	0.0949 ^b^	0.0067 ^a^	<0.0001 ^e^
POLYSACCH FIII	0.7491 ^a^	0.7229 ^a^	0.6994 ^a^	0.7737 ^e^
OLIGOSACCH FIV	0.1025 ^c^	0.4514 ^c^	0.0104 ^c^	0.0103 ^f^
SACCH	>0.9999 ^b^	0.5451 ^b^	0.9510 ^b^	0.2503 ^f^
POLYSACH	0.1345 ^c^	0.0520 ^c^	0.0263 ^c^	0.0211 ^e^
Gallic acid	0.9328 ^c^	0.4472 ^c^	0.4531 ^c^	0.4985 ^e^
Protocatechuic acid	0.0003	0.9639 ^a^	0.1136 ^a^	0.0372 ^e^
BENZOIC ACIDS	0.0303 ^a^	0.7327 ^a^	0.1632 ^a^	0.0059 ^e^
Caftaric acid	0.5445 ^c^	0.0131 ^a^	0.0123 ^a^	0.0405 ^e^
*cis*-Coutaric acid	0.0069 ^a^	0.0342 ^c^	0.0026 ^a^	0.0494 ^g^
*trans*-Coutaric acid	0.7551 ^c^	0.0475 ^c^	0.0029 ^c^	0.0782 ^g^
TART HYDROXYCINNAMATES	0.4588 ^c^	0.0125 ^a^	0.0098 ^a^	0.0962 ^g^
*cis*-caffeic acid	0.3428 ^a^	0.2409 ^a^	0.0315 ^a^	0.1074 ^e^
*trans*-caffeic acid	0.1484 ^a^	0.3704 ^a^	0.0368 ^a^	0.6203 ^e^
*p*-coumaric acid	0.8694 ^c^	0.2059 ^c^	0.2797 ^c^	0.6203 ^e^
FREE HYDROXYCINNAMIC ACIDS	0.2735 ^a^	0.1980 ^a^	0.0289 ^a^	0.3548 ^e^
TOTAL HYDROXYCINNAMATES	0.3007 ^a^	0.0162 ^c^	0.0059 ^a^	0.1022 ^g^
Catechin	0.3376 ^a^	0.6668 ^a^	0.2944 ^a^	0.8766 ^e^
Epicatechin	0.5140 ^a^	0.7026 ^a^	0.3063 ^a^	0.0992 ^e^
Procyanidin B1	0.0017 ^b^	0.1140 ^b^	0.1369 ^b^	0.3433 ^d^
FLAVANOLS	0.1874 ^a^	0.6224 ^a^	0.5008 ^a^	0.3348 ^e^
Tyrosol	<0.0001 ^c^	0.0444 ^c^	0.0551 ^c^	0.3455 ^e^
Tryptophol	0.1498 ^a^	0.2394 ^b^	0.0661 ^a^	0.4605 ^e^
PHENOLIC ALCOHOLS	0.0001 ^a^	0.0236 ^c^	0.0153 ^c^	0.1661 ^e^
*cis*-Piceid	0.1454 ^b^	0.6989 ^b^	0.5441 ^b^	0.6034 ^f^
*trans*-resveratrol	0.3723 ^b^	0.3854 ^b^	0.6744 ^b^	0.9965 ^d^
STILBENES	0.2995 ^b^	0.2998 ^b^	0.6159 ^b^	0.9820 ^d^
Astilbin	0.1204 ^c^	0.3143 ^c^	0.1972 ^c^	0.0449 ^d^
Quercetin-3-Glucoside	0.0511 ^b^	0.0300 ^b^	0.0050 ^b^	0.0450 ^h^
Quercetin	0.0223 ^c^	0.4062 ^b^	0.4522 ^b^	0.2150 ^f^
FLAVONOLS	0.0062 ^c^	0.0557 ^b^	0.0336 ^c^	0.0683 ^h^

^a^ Unpaired Welch’s *t*-test. ^b^ Mann–Whitney test. ^c^ Unpaired Lognormal Welch’s *t*-test. ^d^ Kruskal–Wallis test. ^e^ Unpaired ordinary one-way ANOVA, Tukey’s multiple comparisons test. ^f^ Lognormal unpaired ordinary one-way ANOVA. ^g^ Brown–Forsythe and Welch ANOVA test. ^h^ Lognormal Brown–Forsythe and Welch ANOVA test.

## Data Availability

The original contributions presented in this study are included in the article/Supplementary Material. Further inquiries can be directed to the corresponding authors.

## Supplementary Materials

The following supporting information can be downloaded at: https://www.mdpi.com/article/10.3390/foods15101735/s1, Table S1: Sample code, origin (production valley), vintage and closure type, of the commercial Chardonnay wines analyzed; Table S2: Bioclimatic characterization of vineyard regions during the 2022–2023 and 2023–2024 growing seasons in Chile; Table S3: Basic parameters of the commercial Chardonnay wines analyzed; Figure S1: Geographic distribution of sampled Chardonnay wines across Chilean viticultural regions; Figure S2: Heatmap of Spearman correlation coefficients among phenolic compounds, polysaccharide fractions, and color variables in commercial Chardonnay wines; Figure S3: Conceptual framework illustrating the multifactorial interactions among geographic origin, grape composition at harvest, winemaking practices, and closure-associated bottle evolution contributing to the chemical composition and potential sensory properties of commercial Chardonnay wines.

## Abbreviations

The following abbreviations are used in this manuscript:

ANOVA	Analysis of variance
CIELab	Commission Internationale de l’Éclairage color space
C*	Chroma
h*	Hue angle
L*	Lightness
a*	Red–green coordinate
b*	Yellow–blue coordinate
FI	High molecular weight polysaccharide fraction
FII	Medium molecular weight polysaccharide fraction
FIII	Low molecular weight polysaccharide fraction
FIV	Oligosaccharide fraction
POLYSACH	Total polysaccharides (FI + FII + FIII)
SACCH	Total saccharides (FI + FII + FIII + FIV)
HPLC–DAD	High-performance liquid chromatography with diode array detection
HPSEC–RID	High-performance size exclusion chromatography with refractive index detection
GAE	Gallic acid equivalents
GDD	Growing degree days
OIV	International Organisation of Vine and Wine
PCA	Principal component analysis
UV–Vis	Ultraviolet–visible spectroscopy
SD	Standard deviation
