# Supplementary material for: Phenolic, Polysaccharide, and Color Variability in Chilean Commercial Chardonnay Wines: Influence of Closure Type, Geographic Origin, and Vintage
Source: Foods. 2026 May 14;15(10):1735. doi: 10.3390/foods15101735 (PMC13205873; doi:10.3390/foods15101735)
Supplement: Supplementary file 1 [file foods-15-01735-s001.zip › foods-4299398-supplementary.pdf]

foods

MDPI

Supplemental data

Table S1. Sample code, origin (production valley), vintage and closure type, of the commercial Chardonnay wines analyzed.

Code	Origin (Valley)	Vintage	Location	Closure Type	Ageing
CL23-01	Valle Central	2023	inland	Screw cap	Not specified on label
CL23-02	Valle de Aconcagua Costa	2023	coastal	Screw cap	Not specified on label
CL23-03	San Antonio	2023	coastal	Screw cap	10 months/French barrels
CL23-04	Valle de Limarí	2023	coastal	Cork	Not specified on label
CL23-05	Valle de Leyda	2023	coastal	Screw cap	Not specified on label
CL23-06	Valle de Aconcagua	2023	inland	Screw cap	Not specified on label
CL23-07	Valle de Cachapoal	2023	inland	Screw cap	Not specified on label
CL23-08	Valle Central	2023	inland	Screw cap	Not specified on label
CL23-09	Valle de Leyda	2023	coastal	Screw cap	8 months/French barrels
CL23-10	Valle de Limarí	2023	coastal	Cork	Not specified on label
CL23-11	Valle de Casablanca	2023	coastal	Cork	10 months/French barrels
CL23-12	Valle de Casablanca	2023	coastal	Cork	Not specified on label
CL23-13	Valle de Limarí	2023	coastal	Cork	Not specified on label
CL23-14	Valle de Limarí	2023	coastal	Cork	Not specified on label
CL23-15	Valle de Malleco	2023	inland	Cork	Not specified on label
CL24-01	Valle de Limarí	2024	coastal	Screw cap	Not specified on label
CL24-02	Valle de Aconcagua	2024	inland	Cork	Not specified on label
CL24-03	Valle de Casablanca	2024	coastal	Cork	6 months/French barrels
CL24-04	Valle de Casablanca	2024	coastal	Cork	Not specified on label
CL24-05	Valle de Casablanca	2024	coastal	Screw cap	Not specified on label
CL24-06	Valle de Casablanca	2024	coastal	Cork	Not specified on label
CL24-07	Valle de Leyda	2024	coastal	Screw cap	Not specified on label
CL24-08	Valle de Bío Bío	2024	inland	Cork	Not specified on label
CL24-09	Valle Central	2024	inland	Screw cap	Not specified on label
CL24-10	Valle Central	2024	inland	Screw cap	Not specified on label
CL24-11	Valle Central	2024	inland	Screw cap	Not specified on label
CL24-12	Valle de Colchagua	2024	inland	Screw cap	Not specified on label
CL24-13	Valle de Colchagua	2024	inland	Screw cap	Not specified on label
CL24-14	Valle de Colchagua	2024	inland	Screw cap	Not specified on label
CL24-15	Valle de Colchagua	2024	inland	Screw cap	Not specified on label

Abbreviations: Cork = natural cork stopper; Screw cap = aluminum closure.

Table S2. Bioclimatic characterization of vineyard regions during the 2022–2023 and 2023–2024 growing seasons in Chile.

Region	Valleys included	Growing season	Mean temperature (°C)	Growing Degree Days (GDD, °C)	Precipitation (mm)	Heatwaves (no. events)	Frost events (no.)	Water status
Coquimbo	Limarí	2022–2023	17.8–19.5	1350–1550	15–35	2–4	0–1	Severe drought
		2023–2024	17.5–19.0	1300–1500	25–60	1–3	0–1	Severe drought (slightly improved)
Valparaíso (Coastal)	Casablanca, San Antonio, Leyda	2022–2023	16.5–18.0	1200–1400	40–85	2–3	1–3	Moderate water deficit
		2023–2024	16.0–17.5	1150–1350	80–140	1–2	2–4	Improved water availability
Aconcagua (Interior + Costa	Aconcagua Valley, Aconcagua Costa	2022–2023	18.5–21.0	1500–1750	25–70	3–5	0–2	Moderate drought
		2023–2024	18.0–20.0	1450–1700	70–130	2–4	1–3	Improved water status
Central Valley (RM–O'Higgins–Maule)	Central Valley, Cachapoal, Colchagua	2022–2023	19.0–22.5	1600–1900	30–80	4–6	0–2	Moderate to severe drought
		2023–2024	18.5–21.5	1550–1850	100–200	2–4	1–3	Good water availability
Biobío	Biobío Valley	2022–2023	16.0–18.5	1150–1350	120–250	1–2	1–3	Adequate
		2023–2024	15.5–18.0	1100–1300	180–320	0–2	2–4	High water availability
Araucanía	Malleco Valley	2022–2023	15.5–17.5	1050–1250	150–300	0–1	2–4	Adequate
		2023–2024	15.0–17.0	1000–1200	220–380	0–1	3–5	High water availability

Note: Bioclimatic variables correspond to the vegetative period (September–April) and are expressed as regional ranges to account for intra-valley variability. Data were obtained from Oficina de Estudios y Políticas Agrarias (ODEPA) harvest reports, Dirección Meteorológica de Chile (DMC), and Red Agrometeorológica Nacional (AGROMET) datasets [S1-S4].

Table S3. Basic parameters of the commercial Chardonnay wines analyzed.

Code	% Vol.	pH	T.A. (g/L)
CL23-01	14.0	3.15	3.48
CL23-02	13.5	3.13	3.79
CL23-03	13.5	3.23	3.82
CL23-04	13.5	3.30	4.14
CL23-05	12.5	3.00	4.04
CL23-06	13.5	3.32	3.48
CL23-07	13.5	3.24	3.48
CL23-08	13.5	3.17	4.05
CL23-09	13.0	3.44	3.74
CL23-10	13.5	3.20	4.17
CL23-11	13.0	3.33	3.41
CL23-12	13.5	3.29	4.03
CL23-13	13.5	3.28	4.31
CL23-14	13.0	3.08	5.61
CL23-15	13.0	3.18	4.39
CL24-01	13.0	3.07	4.41
CL24-02	13.5	3.19	4.40
CL24-03	13.5	3.14	3.85
CL24-04	14.0	3.13	4.31
CL24-05	13.5	3.17	3.77
CL24-06	13.0	3.32	3.68
CL24-07	13.5	3.17	4.77
CL24-08	13.5	3.41	3.42
CL24-09	12.5	3.20	4.14
CL24-10	12.5	3.31	3.19
CL24-11	12.5	3.19	4.12
CL24-12	13.0	3.12	4.21
CL24-13	13.5	3.16	3.41
CL24-14	13.5	3.20	3.79
CL24-15	14.0	3.06	3.60

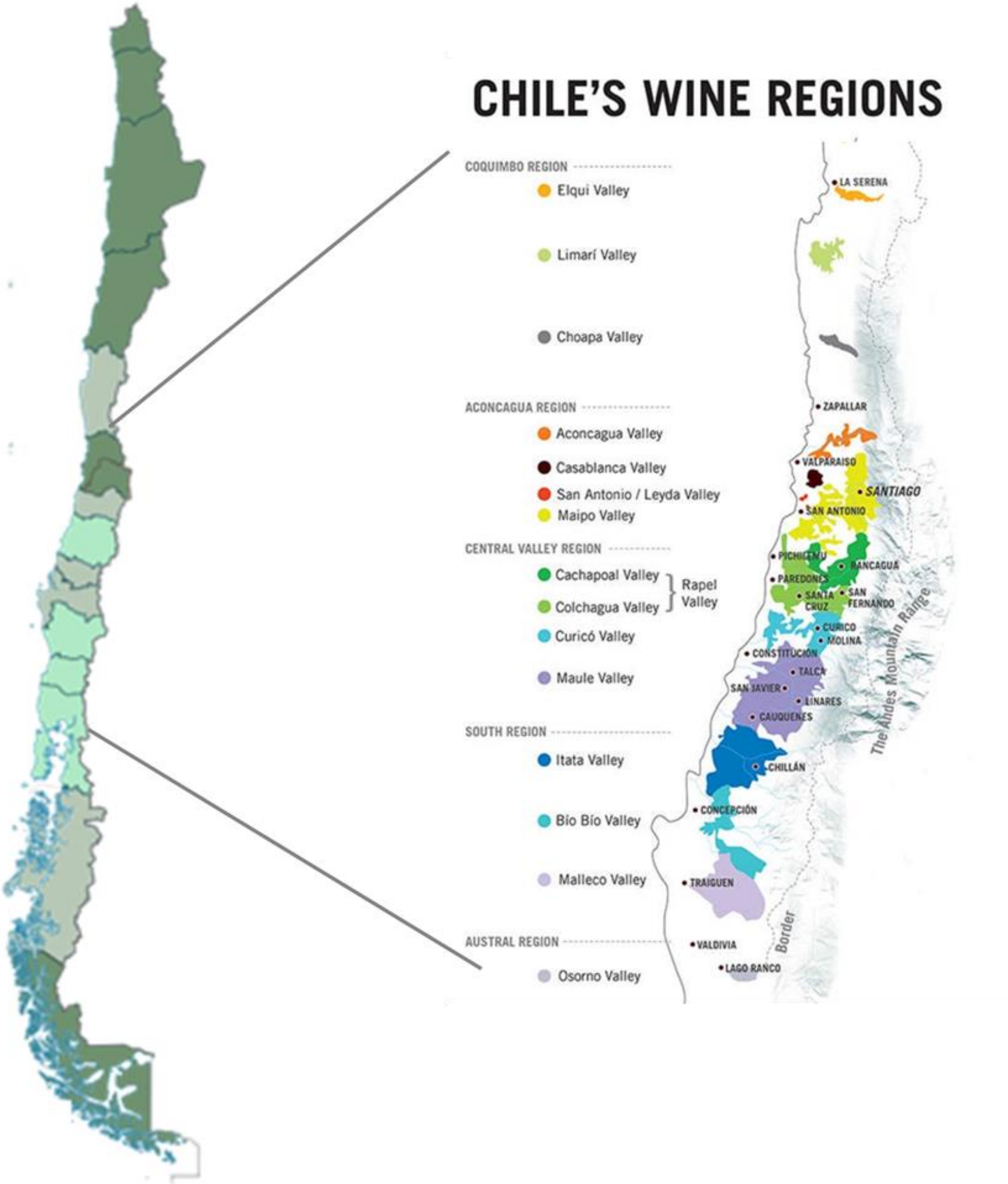

Figure S1. Geographic distribution of sampled Chardonnay wines across Chilean viticultural regions. Referential map showing the main valleys included in the study and a simplified inset illustrating their spatial distribution.

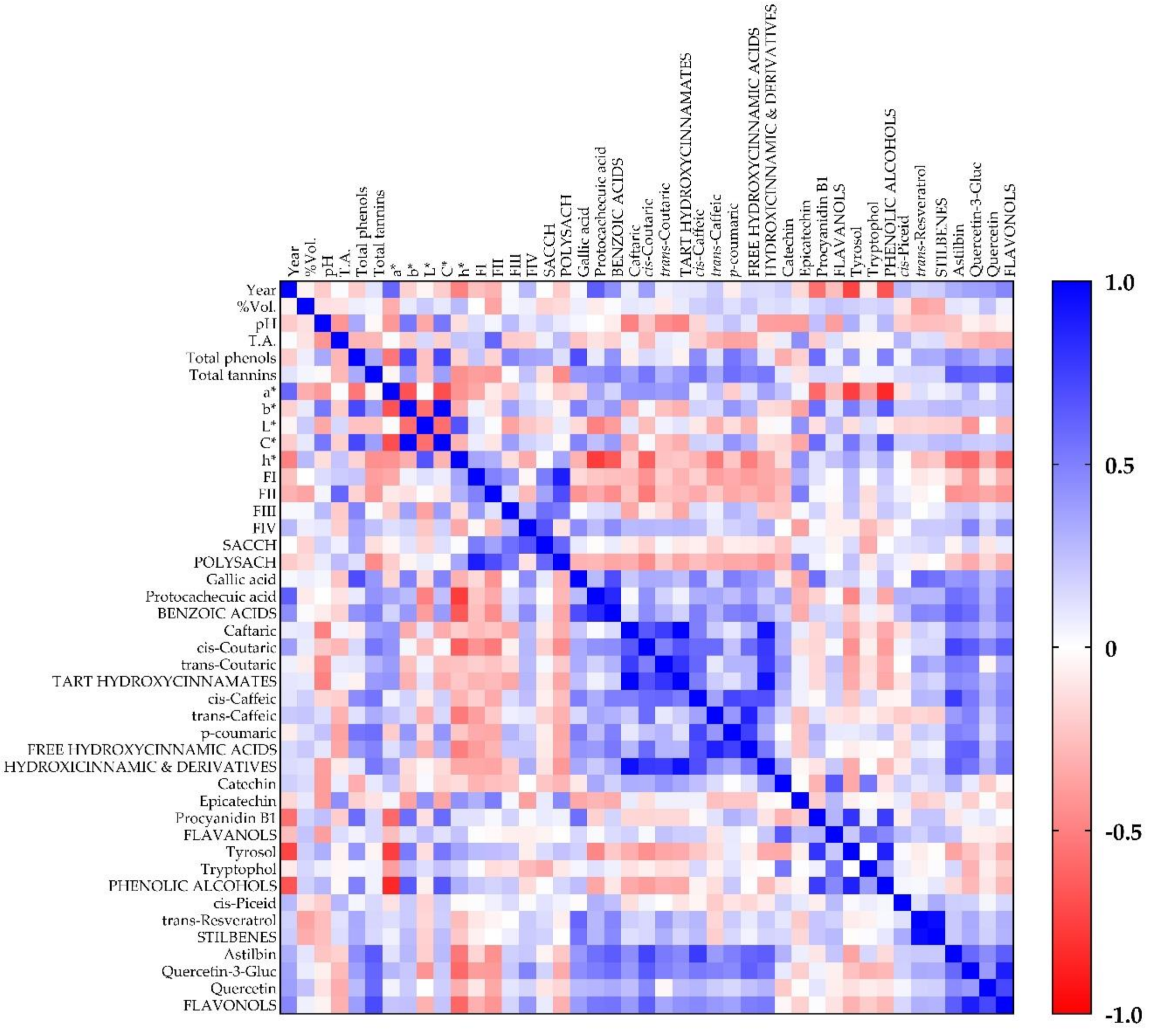

Figure S2. Heatmap of Spearman correlation coefficients among phenolic compounds, polysaccharide fractions, and color variables in commercial Chardonnay wines. Color intensity represents the magnitude and direction of the correlation coefficients. Only significant correlations are displayed according to the criteria defined in the statistical analysis.

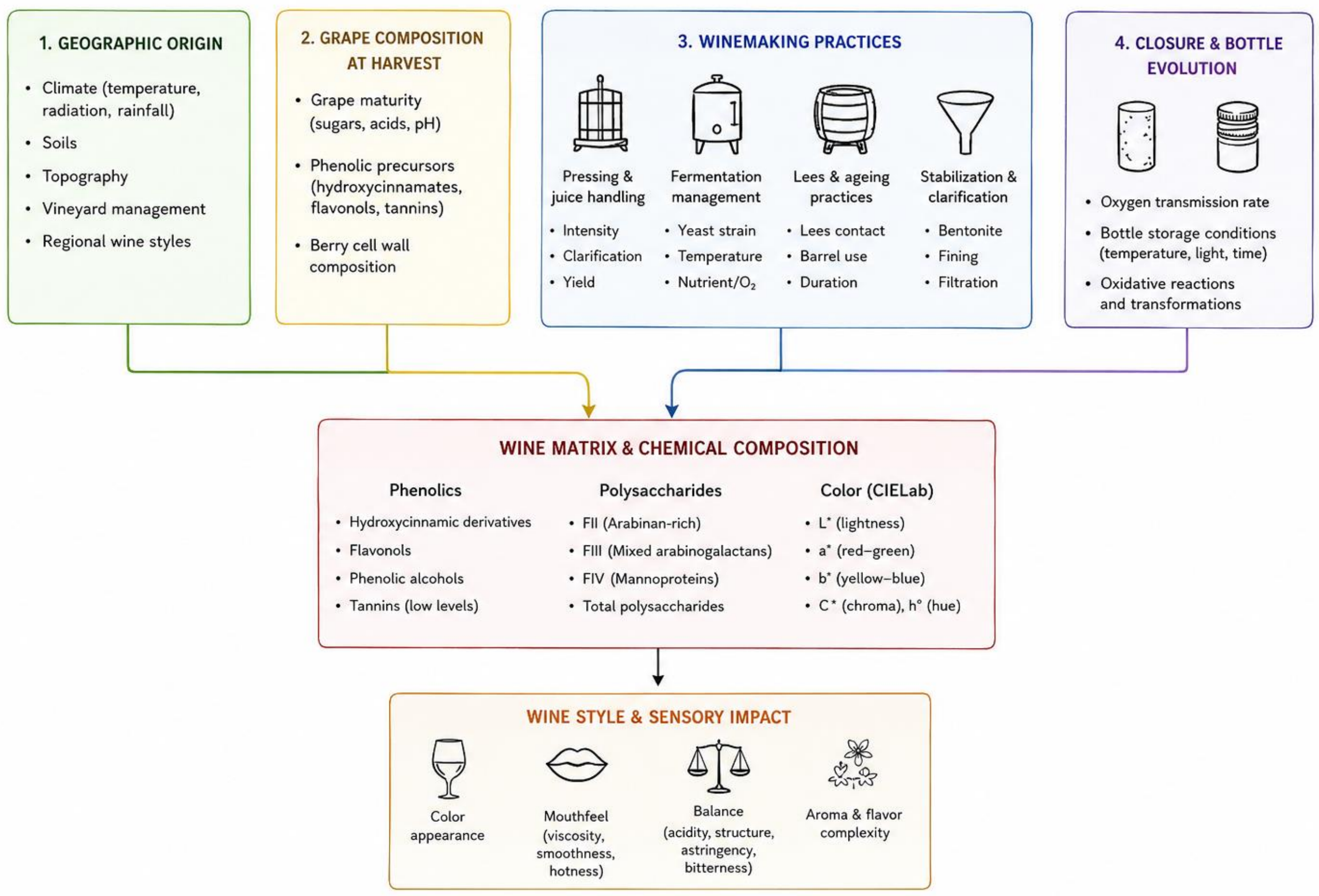

Figure S3. Conceptual framework illustrating the multifactorial interactions among geographic origin, grape composition at harvest, winemaking practices, and closure-associated bottle evolution contributing to the chemical composition and potential sensory properties of commercial Chardonnay wines.

References

S1. Oficina de Estudios y Políticas Agrarias (ODEPA). *Wine Harvest Forecast Report 2023*; Ministry of Agriculture: Santiago, Chile, 2023.

S2. Oficina de Estudios y Políticas Agrarias (ODEPA). *Wine Harvest Forecast Report 2024*; Ministry of Agriculture: Santiago, Chile, 2024.

S3. Dirección Meteorológica de Chile (DMC). *Climatological Reports 2022–2024*; DMC: Santiago, Chile, 2024.

S4. Red Agrometeorológica Nacional (AGROMET). *Agroclimatic Data Records 2022–2024*; INIA: Santiago, Chile, 2024.
